# Cefiderocol Elimination During Peritoneal Dialysis: A Short Report

**DOI:** 10.7759/cureus.99579

**Published:** 2025-12-18

**Authors:** Julian Müller-Kühnle, Bettina Rittershofer, Moritz Schanz, Sebastian Allgäuer, Thomas Muerdter, Severin Schricker

**Affiliations:** 1 Department of General Internal Medicine and Nephrology, Robert Bosch Hospital, Stuttgart, DEU; 2 Department of Anesthesia and Operative Intensive Care Medicine, Robert Bosch Hospital, Stuttgart, DEU; 3 Department of Cardiology and Angiology, Robert Bosch Hospital, Stuttgart, DEU; 4 Department of Chemical Analytics and Synthesis, Dr. Margarete Fischer-Bosch Institute of Clinical Pharmacology, Stuttgart, DEU

**Keywords:** cefiderocol, hemodialysis, peritoneal dialysis, peritoneal dialysis pharmacokinetics, siderophore cephalosporin, therapeutic drug monitoring

## Abstract

Cefiderocol is a siderophore cephalosporin with potent activity against multidrug-resistant Gram-negative pathogens, yet pharmacokinetic data for patients receiving peritoneal dialysis (PD) are lacking. We report the first clinical observation of cefiderocol elimination during PD, complemented by an intra-individual comparison with hemodialysis (HD) and reference measurements from a second patient not receiving dialysis. A 64-year-old man on continuous ambulatory PD received cefiderocol 2 g every eight hours as three-hour infusions. Serial sampling showed serum trough and peak concentrations of 79.8 and 139.9 mg/L. A single PD exchange reduced serum levels by 32% (from 139.9 to 95.6 mg/L) with near-complete equilibration in dialysate (94 mg/L), indicating meaningful peritoneal removal. During subsequent conversion to HD, serum concentrations declined more rapidly, consistent with higher extracorporeal clearance. A second patient with acute-on-chronic kidney injury who did not receive dialysis exhibited the highest concentrations (trough 164.9 mg/L; peak 280.1 mg/L), highlighting the impact of absent extracorporeal elimination. Treatment was clinically successful and well-tolerated in both cases, with no neurotoxicity or other adverse events observed. These findings demonstrate that PD provides moderate but consistent cefiderocol elimination, substantially less efficient than HD, and suggest that therapeutic drug monitoring may help optimize dosing and balance efficacy and safety in PD. Larger studies are warranted to refine dosing recommendations for this population.

## Introduction

Cefiderocol is a siderophore cephalosporin with potent activity against multidrug-resistant Gram-negative pathogens, including carbapenem-resistant *Enterobacterales* and non-fermenters [1]. Pharmacokinetic data exist for patients with normal renal function and for those receiving hemodialysis (HD) [2-4], but no information is available for peritoneal dialysis (PD). This gap hampers dosing recommendations for novel antimicrobials in PD.

We therefore aimed to characterize cefiderocol exposure and extracorporeal removal in a PD patient to provide initial, real-world data to inform dosing considerations in this population. We report the first clinical observation of cefiderocol clearance during PD, including an intra-individual comparison with HD and reference data from a second patient not receiving dialysis.

## Case presentation

A 64-year-old man with end-stage kidney disease from diabetic nephropathy had been maintained on continuous ambulatory peritoneal dialysis (CAPD) since August 2024. He was admitted with parapneumonic pleural empyema requiring thoracotomy and decortication. During prolonged postoperative ventilation, he developed ventilator-associated pneumonia due to extensively drug-resistant (4MRGN) *Klebsiella pneumoniae*, resistant to all standard antibiotics. Cefiderocol was initiated at 2 g every eight hours as three-hour infusions for 10 days, combined with intravenous colistin. PD was briefly interrupted for percutaneous endoscopic gastrostomy placement, necessitating conversion to sustained low-efficiency HD. This clinical sequence enabled an intra-individual comparison of cefiderocol exposure and extracorporeal removal between PD and HD under identical dosing (Table 1).

**Table 1:** Cefiderocol serum concentrations at individual sampling points Concentrations are reported as total drug (mg/L). Dates use day.month format. "During HD" indicates an intradialytic sample. "Post-HD" indicates the first post-dialysis sample. "Serum, 4 hours post-dose" refers to the time since infusion end. Dialysate was sampled during a single PD exchange (2,000 mL, 1.36% low-glucose solution). No established therapeutic ("reference") ranges for cefiderocol serum concentrations are defined in the product label or clinical guidelines. Values are shown as measured. Interpretation should be based on PK/PD target attainment, i.e., the time that unbound concentrations exceed the MIC (fT>MIC) relative to the organism's MIC. Dialysate concentrations have no reference range. HD: hemodialysis; PD: peritoneal dialysis; AKI: acute kidney injury; ESKD: end-stage kidney disease; PK: pharmacokinetics; MIC: minimum inhibitory concentration; fT: fraction of time

Patient	Date/time	Dialysis status	Concentration (mg/L)	Sampling context
1	13.06. 11:30	Pre-HD	72.80	Pre-dialysis trough
1	13.06. 14:00	During HD	65.56	Intradialytic
1	13.06. 23:00	Post-HD	39.61	Post-dialysis
1	16.06. 09:45	PD	79.82	Pre-infusion trough
1	16.06. 10:40	PD	139.93	Peak (post-infusion)
1	16.06. 14:30	PD	95.63	Serum (4h post-dose)
1	16.06. 14:30	PD	94.04	Dialysate
2	16.06. 13:30	None (AKI/ESKD)	164.89	Trough (stable renal function)
2	16.06. 15:00	None (AKI/ESKD)	280.10	Peak (post-infusion)

**Figure 1:**
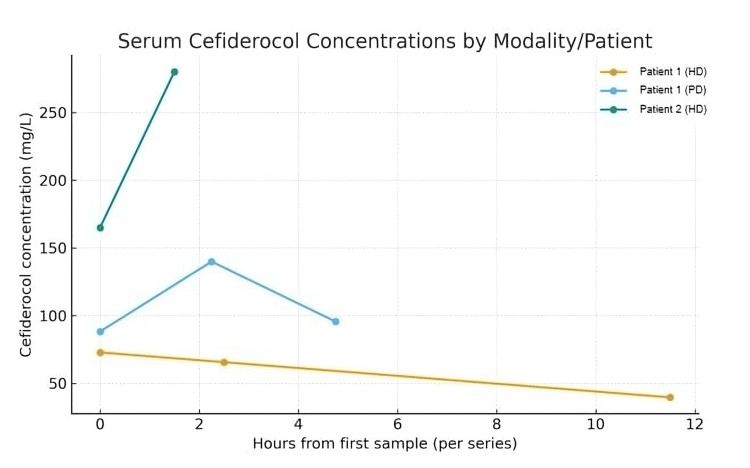
Serum cefiderocol concentrations by modality and patient Concentration-time profiles plotted as hours from the first sample within each series for Patient 1 during HD (13 June), Patient 1 during PD (16 June), and Patient 2 (history of HD before cefiderocol initiation) before and after infusion (16 June). Symbols denote individual samples. Higher and sustained concentrations during PD indicate minimal clearance, whereas HD shows decline. HD: hemodialysis; PD: peritoneal dialysis

Serial serum samples were obtained immediately before and after cefiderocol infusions, during PD exchanges, and during HD sessions; during one PD exchange, dialysate was also sampled for drug concentration. While on PD, serum concentrations were 79.8 mg/L at trough and 139.9 mg/L at peak. A single PD exchange with 2,000 mL of balanced low-glucose solution at 1.36% reduced the serum concentration by 32%, from 139.9 to 95.6 mg/L, and the dialysate concentration reached 94 mg/L, indicating near-complete equilibration and clinically meaningful peritoneal removal (Table 1). After transition to HD, serum levels declined more rapidly, from 72.8 mg/L before dialysis to 65.6 mg/L during dialysis and 39.6 mg/L after dialysis, consistent with higher extracorporeal clearance (Figure 1). The infection resolved under therapy, and cefiderocol was well-tolerated without adverse events.

A second patient, a 71-year-old woman with a history of bilateral lung transplantation for progressive fibrosing interstitial lung disease, presented with sepsis caused by extensively drug-resistant (4MRGN) *Klebsiella pneumoniae*. She had previously required temporary HD for acute-on-chronic kidney injury, but renal function had recovered before cefiderocol initiation, with an estimated glomerular filtration rate of 34-50 mL/min/1.73 m². Cefiderocol 2 g every eight hours as three-hour infusions was administered for nine days alongside other antimicrobial agents. Two serum samples showed the highest observed concentrations, 164.9 mg/L at trough and 280.1 mg/L at peak, providing reference measurements for impaired yet dialysis-independent renal function (see Table 1 and Figure 1). Treatment was clinically successful and well-tolerated.

Cefiderocol concentrations in serum and dialysate were quantified using a validated liquid chromatography-tandem mass spectrometry (LC-MS/MS) assay established for routine therapeutic drug monitoring. Results are reported as measured values without pharmacokinetic modeling.

## Discussion

To our knowledge, this report provides the first clinical description of cefiderocol clearance during PD. The intra-individual comparison with HD shows that PD achieves meaningful, albeit slower, drug elimination. A single PD exchange removed approximately one-third (32%) of circulating cefiderocol, with near-complete equilibration between serum and dialysate, indicating efficient peritoneal transfer of cefiderocol in this patient. In contrast, HD rapidly reduced serum concentrations, consistent with its substantially greater solute clearance capacity.

Data from a nondialysis patient confirmed sustained high cefiderocol levels in the absence of extracorporeal elimination, underscoring the impact of renal replacement modality on systemic exposure. These very high trough and peak concentrations occurred in a patient with impaired but dialysis-independent renal function (estimated glomerular filtration rate 34-50 mL/min/1.73 m²).

Population pharmacokinetic analyses in individuals with normal renal function report a geometric mean total clearance of approximately 4.7 L/h and an elimination half-life of 2-3 hours, with cefiderocol being eliminated predominantly unchanged via the kidneys [2,3]. The marked discrepancy between these reference values and the sustained high concentrations in our nondialysis patient is therefore consistent with substantially reduced intrinsic renal clearance in addition to the lack of extracorporeal removal.

Cefiderocol is primarily excreted renally, and total clearance correlates with kidney function [4]. Whereas HD efficiently removes hydrophilic antibiotics with low to moderate protein binding [5,6], PD relies on slower solute transfer and smaller dialysate volumes [7]. Our observations indicate that PD contributes to cefiderocol elimination, but considerably less efficiently than HD.

From a therapeutic standpoint, these findings are relevant in two ways. First, PD patients such as the one described here may experience sustained cefiderocol exposure with only moderate removal per exchange, potentially prolonging effective concentrations. Second, the high trough levels observed in the nondialysis patient (>150 mg/L) fall within ranges where cephalosporin-associated neurotoxicity has been reported [8,9]. Although no adverse events occurred, these results highlight the potential utility of therapeutic drug monitoring to balance efficacy and safety during prolonged therapy, especially in patients with advanced renal impairment and limited or absent extracorporeal clearance options.

Strengths of this report include serial sampling, intra-individual comparison across dialysis modalities, and direct analysis of dialysate. Limitations are the small sample size, the absence of formal pharmacokinetic modeling, limited sampling in the second patient, and the lack of a recent peritoneal equilibration test, which precludes formal classification of the peritoneal transport type and may limit generalizability to PD patients with different membrane characteristics.

## Conclusions

In this PD patient, cefiderocol demonstrated moderate extracorporeal clearance, with approximately 32% of circulating drug removed during a single exchange and near-complete equilibration in dialysate. Hemodialysis achieved substantially greater clearance, whereas the absence of dialysis in a second patient with impaired but dialysis-independent renal function was associated with persistently elevated concentrations. Collectively, these observations suggest that PD provides consistent but limited cefiderocol elimination and that dosing in PD should account for moderate drug removal while still anticipating high systemic exposure. Until larger studies define PD-specific dosing recommendations, therapeutic drug monitoring may support individualized cefiderocol therapy in this population and in patients with advanced renal impairment and limited extracorporeal clearance options.
